# Intraoperative Autologous Platelet-Rich Stroma Injection as Add-On to Fistula Curettage and Closure of the Internal Orifice Demonstrates a Favorable Outcome in Long-Term in Patients Suffering from Therapy-Refractory Perianal Fistulizing Crohn’s Disease

**DOI:** 10.1093/ibd/izaf011

**Published:** 2025-05-24

**Authors:** Michiel T J Bak, Annemarie C de Vries, Caroline D M Witjes, Jeanine H C Arkenbosch, Roy S Dwarkasing, Joris A van Dongen, Gwenny M Fuhler, Willem Rudolph Schouten, Christien Janneke van der Woude, Oddeke van Ruler

**Affiliations:** Department of Gastroenterology and Hepatology, Erasmus University Medical Center, Rotterdam, The Netherlands; Department of Gastroenterology and Hepatology, Erasmus University Medical Center, Rotterdam, The Netherlands; Department of Surgery, IJsselland Hospital, Capelle aan den IJssel, The Netherlands; Department of Surgery, Erasmus University Medical Center, Rotterdam, The Netherlands; Department of Gastroenterology and Hepatology, Erasmus University Medical Center, Rotterdam, The Netherlands; Department of Radiology, Erasmus University Medical Center, Rotterdam, The Netherlands; Department of Plastic, Reconstructive and Hand Surgery, University Medical Center Utrecht, Utrecht University, Utrecht, The Netherlands; Department of Gastroenterology and Hepatology, Erasmus University Medical Center, Rotterdam, The Netherlands; Department of Surgery, IJsselland Hospital, Capelle aan den IJssel, The Netherlands; Department of Gastroenterology and Hepatology, Erasmus University Medical Center, Rotterdam, The Netherlands; Department of Gastroenterology and Hepatology, Erasmus University Medical Center, Rotterdam, The Netherlands; Department of Surgery, IJsselland Hospital, Capelle aan den IJssel, The Netherlands; Department of Surgery, Erasmus University Medical Center, Rotterdam, The Netherlands

**Keywords:** perianal fistula, Crohn’s disease, platelet-rich stroma, stromal vascular fraction, platelet-rich plasma, autologous, cell therapy

## Abstract

**Background:**

An injection with autologous platelet-rich stroma (PRS), a combination of stromal vascular fraction and platelet-rich plasma, as an add-on to fistula curretage and closure of the internal orifice proved to be safe and feasible for the treatment of patients with treatment-refractory perianal fistulizing Crohn’s disease (pCD). This study aimed to assess the long-term outcomes in patients with pCD treated with autologous PRS injection.

**Methods:**

Adult patients with therapy-refractory pCD (failure to anti-tumor necrosis factor [TNF] therapy and/or fistula surgery), who underwent fistula curettage, closure of the internal fistula orifice, and autologous PRS injection in a Dutch tertiary referral center were included in an earlier conducted pilot study (*n* = 25). The primary outcome was complete clinical closure at long-term follow-up (closure of all treated external opening[s]). Secondary outcomes were partial clinical closure (closure of ≥1 treated external opening[s]), radiologic healing (fibrotic fistula tract on magnetic resonance imaging), and recurrence.

**Results:**

The majority of the patients were female (56%) (mean age 34.4 years [standard deviation – SD: 0.9], and mean follow-up 3.7 years [SD: 0.6]). The treatment-refractory character of the study cohort was displayed by the high rate of patients with ≥1 external opening (60%), prior exposure to an anti-TNF agent (92%), TOpClass classification system ≥ class 2b (36%), and the low rate of patients who underwent prior surgical interventions aimed at fistula closure (12%). During long-term follow-up, complete clinical closure was achieved in 88%. Partial clinical closure was achieved in all patients. Radiologic healing was achieved in 75% of the patients. Recurrence was reported in 8% of the patients who achieved prior clinical closure. No recurrences were observed in patients with radiologic healing. Seventeen unplanned re-interventions were reported in nine patients (36%), predominantly for residual fistulizing disease and in patients with severe therapy-refractory pCD (TOpClass classification system ≥ class 2b) at the time of inclusion.

**Conclusion:**

Additional PRS injection, fistula curettage, and closure of the internal orifice is a promising therapy for patients with (treatment-refractory) pCD and could improve clinical and radiologic healing rates. In addition, low recurrence rates were observed. Future randomized research is warranted in order to assess the effectiveness and positioning of PRS in the field of pCD.

**Clinical trial registration:**

NL8417

**Key Messages**

**What is already known?**

An injection with autologous platelet-rich stroma, a combined product of a stromal vascular fraction and platelet-rich plasma, as an add-on to fistula curettage and closure of the internal orifice, has proved to be safe, feasible, and beneficial on short-term for therapy-refractory perianal fistulizing Crohn’s disease (pCD). However, long-term outcomes of this treatment are awaited.

**What is new here?**

Complete clinical closure and partial clinical closure were eventually achieved in 88% and 100% of the patients including the necessity for unplanned re-interventions in 36% of the patients. Subsequently, radiologic healing is achieved in 75% of the patients. Low recurrence rates after clinical closure were observed (8%).

**How can this study help patient care?**

Injection of autologous platelet-rich stroma (PRS), fistula curettage, and closure of the internal orifice is a promising treatment for therapy-refractory pCD. Future randomized research is necessary to assess the effects and position of autologous PRS in the field of pCD.

## Introduction

Perianal fistulas are common in patients with Crohn’s disease (CD) as approximately 20% of the patients with CD will develop a perianal fistula during their disease course.^1^ Perianal fistulizing CD (pCD) is associated with a high disease burden, an impaired quality of life, and an increase in health-related costs.^2^ pCD is often complex to treat and recurrent in up to 40% of the patients.^3,4^ In addition, long-term remission of complex fistulas in pCD was only reported in 37%.^3^

Guidelines consider a combination of anti-tumor necrosis factor (TNF) agents and surgical interventions aimed at fistula closure as the most effective treatment for pCD in surgically amendable patients, which was recently confirmed by a randomized controlled trial.^5–8^ As perianal fistulas in pCD are often classified as complex (involving the upper two-thirds of the external sphincter and/or with multiple tracts), sphincter-preserving procedures are preferred to achieve fistula closure. However, these procedures fail in 40%-50% of the patients with pCD.^9^ In the past years, new therapeutic options with the use of mesenchymal stem cells, derived from either bone marrow or adipose tissue (adipose-derived stromal cells [ASCs]), have been developed in order to improve fistula healing.^10^ Subsequently, promising outcomes have been reported for both ex vivo allogenic and autologous ASCs.^11–14^ However, recently uncertainties on the effects of adipose-derived allogenic ASCs have been raised due to the conflicting outcomes in the ADMIRE phase 3 trials.^11,14,15^

Platelet-rich stroma (PRS), a combination of stromal vascular fraction (SVF) and platelet-rich plasma (PRP), was introduced in our center for the treatment of complex fistula (transsphincteric cryptoglandular fistula, treatment-refractory Crohn’s related perianal fistula, and rectovaginal fistula).^16,17^ The use of autologous PRS offers several advantages over the treatment with allogenic and autologous ASCs treatment such as the significantly lower costs as compared to cultured or enzymatic obtained ASCs and the possibility to harvest and prepare PRS intra-operatively during a single procedure. Furthermore, the preservation of the extracellular matrix in mechanically isolated adipose-derived SVF is important due to its vital role in the regenerative properties of SVF.^18^

The use of an autologous PRS injection as an add-on to surgical treatment proved to be feasible without (serious) adverse events in patients with transsphincteric cryptoglandular fistula or treatment-refractory pCD.^16,17^ Furthermore, beneficial outcomes have been described in both patients groups. In patients with treatment-refractory pCD, complete clinical closure was achieved in 52% at 12 months whilst radiologic healing was observed in 38% of the patients at 3 months.^16^ However, long-term outcomes of additional autologous PRS injection to fistula curettage and closure of the internal orifice for therapy-refractory pCD are still unknown. Therefore, this present study aimed to assess the long-term clinical and radiologic fistula closure in patients with treatment-refractory pCD treated with an injection of autologous PRS included in this pilot study.

## Materials and Methods

### Study Design

Between March 2019 and August 2020, consecutive patients with pCD undergoing fistula surgery were enrolled in a prospective, non-randomized, pilot cohort study after informed consent in our tertiary referral center. Adult patients with treatment-refractory pCD were included, defined as a perianal fistulizing disease with previously endoscopic or histologically confirmed CD. Treatment-refractory pCD was defined as no fistula closure by at least one of the following medical or surgical treatments: anti-TNFα agents and/or fistula surgery.^16^ Exclusion criteria were active proctitis, presence of associated (not properly drained) pelvic abscess, severe active luminal CD at the time of surgery, immune suppressed status (eg, active human immunodeficiency virus), any hematological disorders and/or oncological event in the past 5 years.

### Operative Technique

**Figure 1.**
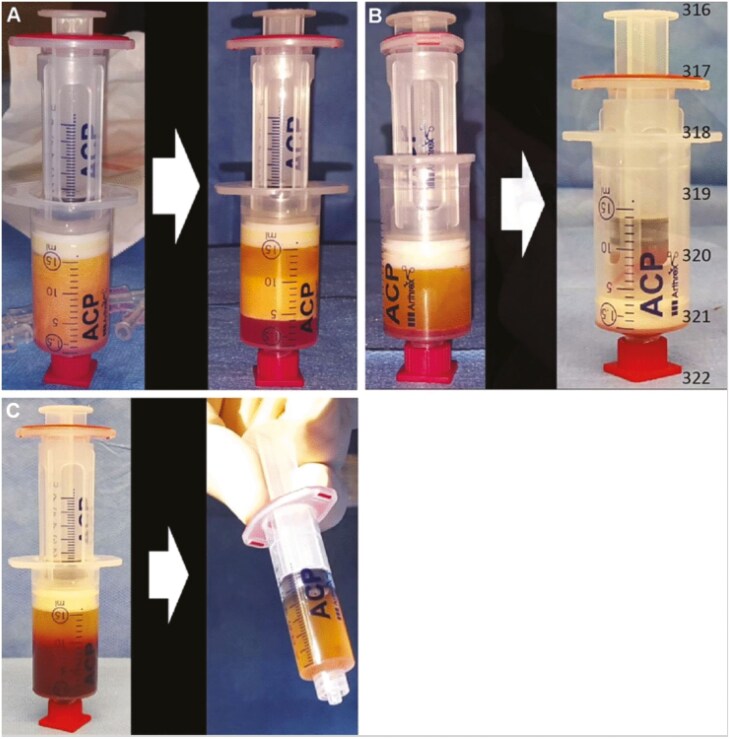
Mechanical fractionation procedure of SVF combined with PRP procedure. Lipoaspirate harvested by liposuction from subcutaneous fatty tissue is centrifuged (5 min, 2500 rpm), resulting in ±10 mL condensed fatty tissue (A), mechanically fractionated and centrifuged again (5 min, 2500 rpm) to obtain 1 mL SVF (B). A venous blood sample (15 mL) is centrifuged (4 min, 1500 rpm) after which 4–5 mL PRP was obtained (C). SVF, stromal vascular fraction; PRP, platelet-rich plasma. Figure and text obtained from Schouten et al.^15^

The operative technique was earlier described by Schouten et al.^17^ A video of the procedure was previously published.^19^ All patients underwent standardized fistula curettage with the closure of the internal orifice combined with the harvesting of subcutaneous lipoaspirate and venous blood sampling to obtain autologous SVF with PRP.^16,17,19^ All procedures were performed by four colorectal surgeons with proctological expertise (W.R.S., O.v.R., M.B.v.O.A., and E.J.D.G). The surgeons were trained by an experienced colorectal surgeon (W.R.S.) and followed the surgical protocol reference guide for the preparation and injection of autologous SVF with PRP.^16,17,19^ Patients underwent induction of general anesthesia followed by endotracheal intubation or spinal anesthesia and received metronidazole (500 mg) and cefazoline (2000 mg) intravenously. A Lone Star retractor (Lone Star Retractor System, Lone Star Medical Products, Inc.) was used to expose the internal orifice(s) of the fistula. At first, the rectal mucosa was assessed by the treating colorectal surgeon to evaluate whether concomitant proctitis was present. The surgeon continued the procedure in case no proctitis was present. In case proctitis was present, the procedure was postponed until the proctitis was treated and resolved. The procedure was only performed in case the above-mentioned in- and exclusion criteria were met. Setons were removed if present. Up to the discretion of the treating surgeon, a temporary drain or Malecot catheter was placed to improve drainage in case of extensive side branches or cavitation was present. The external opening(s) of the fistula was enlarged by coring out to the exterior of the external anal sphincter. If fistula curettage and closure of the internal orifice were considered feasible, lipoaspiration for the harvesting of SVF was initiated. Due to the hypothetical risk of subcutaneous infection, liposuction was performed prior to fistula curettage or by renewed sterilization of the operating field and team. A small paravertebral skin incision was made bilaterally approximately 5 cm cranial to the posterior superior iliac spine. The subcutaneous adipose tissue was infiltrated bilaterally with 40 mL 0.9% saline solution, containing 20 mg/mL lidocaine 2% with 1:100 000 adrenaline added to 500 mL 0.9% saline solution. By aspiration, 15 mL of lipoaspirate was harvested bilaterally with a double syringe system (Arthrex GMBH). Obtained lipoaspirate was centrifuged (2500 rpm, 5 min) using the Rotofix 32A centrifuge (Andreas Hettich, GmbH & Co.KG, Tuttlingen, Germany) and the fistula procedure was initiated. The internal orifice(s) and fistula tract(s) were cleaned and brushed. Centrifugation of the lipoaspirate resulted in three separate fractions: oil, condensed fatty tissue, and aqueous infiltration fluid (Figure 1A). The oil and fluid fractions were removed from the lipoaspirate. Mechanical fractionation of centrifuged lipoaspirate was performed by vigorously passing the lipoaspirate forwards and backwards 30 times through a disposable one-hole fractionator (⌀1.4 mm, luer-to-luer transfer, Tulip). After mechanical fractionation, samples were centrifuged (2500 rpm, 5 min), after which the oily fraction was removed, leaving approximately 1 mL SVF (Figure 1B).^20^ Simultaneously, 15 mL of whole blood was centrifuged (1500 rpm, 4 min), after which 4–5 mL PRP was obtained from the upper layer (plasma) (Figure 1C). A total of approximately 1 mL SVF and 5 mL PRP were combined in one syringe to form PRS. Platelet-rich stroma was injected into the tissue surrounding the curated internal orifice(s) within approximately 2 mm, and into all quadrants of the fistula wall along the fistula tract(s) in several micro-blebs. Subsequently, the internal orifice was closed full-thickness with absorbable sutures 2/0 Vicryl (Ethicon, Inc.).

### Clinical Data Collection

Data were collected at preoperative visits, at the time of fistula surgery, regular scheduled outpatient follow-up visits (6 weeks, 3–6 to 12 months postoperatively), and, if available, long-term follow-up visits up to October 2023 (end of study). Collected data consisted of patient-related characteristics (eg, age and smoking), disease-related characteristics (eg, medication exposure at the time of surgery and pCD disease duration), surgical characteristics, and postoperative course (eg, necessity for a re-intervention). To assess the severity of pCD at the time of surgery, the classification of the TOpCLASS consortium (ie, TOpClass classification system) was assessed retrospectively, by approximation, by an experienced colorectal surgeon (O.R.), blinded for disease outcomes and the potential need for (unplanned) re-interventions, based on chart review and preoperative MRI.^21^

### Outcomes

The primary endpoint was complete clinical closure, defined as complete closure of all treated external opening(s) without discharge at physical examination at the last documented follow-up visit. Secondary endpoints included (I) partial clinical closure, defined as closure of ≥1 treated external opening(s) combined without discharge of these openings at physical examination and (II) radiologic healing, defined as the complete absence of fluid-containing fistula tracts with the absence of T2-weighted (T2w) hyperintense signal at the location of the treated fistula(s) at the last available magnetic resonance imaging (MRI) and (III) recurrence (reopening of the external opening after prior clinical closure and/or radiologic healing) and the need for re-intervention(s).

### Follow-Up

Regular outpatient follow-up visits were scheduled for all patients up to 1 year postoperatively (6 weeks, 3, 6, 12 months postoperatively). Three months after the procedure, repeated treatment with additional PRS injection(s) was considered in patients with clinical and/or radiologic residual fistulizing disease. In case complete clinical and/or radiologic closure was achieved, patients were invited to contact and/or visit the outpatient clinic annually up to 3 years postoperatively. All patients with complete clinical closure were invited for an additional MRI to examine whether radiologic healing was achieved. In case complete clinical and/or radiologic healing was not achieved within one year, further treatment and follow-up visits (including repeat MRI) were discussed by the treating surgeon. At each outpatient follow-up visit, fistula-related complaints (eg, fecal incontinence and soiling) were assessed and a physical examination was conducted to assess the closure of the external opening(s).

### MRI Evaluation

Postoperative MRI of the fistula was performed at 3 months postoperatively and/or during follow-up on case-by-case basis, and centrally assessed by an experienced abdominal radiologist (R.D.), blinded for the clinical outcomes. Pelvic MRI was performed prior to the operative procedure using a 1.5T system with a four-channel phased array coil. Field of view consisted of the lower pelvis, perineum, and skin area with a full display of the anus and lower-mid rectum. The MRI protocol consisted of T2w sequences with and without fat saturation in three planes: axial, sagittal, and coronal. Fistulas were identified based on hyperintens (white) signal. The pre- and post-operative MRIs were in random order.

The modified van Assche index was used to assess the radiologic severity of pCD and included the following items: fistula extension, hyperintensity on T2w images, rectal wall involvement, presence of inflammatory mass, and the dominant feature of the fistula tract.^22^

### Statistical Analyses & Ethical Consideration

Descriptive statistical analysis (frequency, percentage, mean, standard deviation [SD], median, and interquartile range [IQR]) was used to describe the research sample. Categorical variables were quoted as the number and percentage. Continuous variables were tested for normality using the Kolmorgorov-Smirnov test. Normal distributed variables were presented as mean and SD, whilst non-normal distributed variables were presented as median and IQR. To compare the paired pre- and postoperative modified van Assche indices, the Wilcoxon-rank test was used. The time to event, defined as the time from first PRS injection to event (ie, complete clinical and partial clinical closure) was assessed using survival analysis and was displayed with Kaplan-Meier curves. As radiologic healing was assessed at the last available MRI, no time to event **analysis** for this outcome was assessed. A *P*-value < .05 was considered statistically significant. Data analysis was performed using SPSS (version 21, IBM, Chicago, IL).

This study was performed in accordance with the 2008 Declaration of Helsinki. This study was reviewed and approved by the local Medical Ethical Committee (trial number NL8417). Written informed consent was obtained from patients included in the study.

## Results

All consecutive patients (*n* = 25), included in the earlier conducted pilot study, with treatment-refractory pCD were included in this long-term follow-up study (mean follow-up time 3.7 years [SD: 0.6]). Baseline characteristics are displayed in Table 1. The majority of the patients were female (56%, *n* = 14) with a median pCD duration of 4 years (IQR 2–8). More than one external opening was present in 60% of the patients. About 92% of the patients were treated with an anti-TNF agent prior to surgery. In retrospect, the TOpClass classification system at the time of surgery comprised classes 2a, 2b, 2c-I, and 2c-ii in 64%, 28%, 4%, and 4%, respectively. Three patients (12%) underwent prior surgical interventions aimed at fistula closure. Short-term outcomes were earlier reported (partial and complete closure rate at 12 months: 80% and 52%; radiologic healing at 3 months: 38%).^16^

**Table 1.** Baseline characteristics (*n* = 25).

Variables	Values
Female sex, *n*(%)	14 (56)
Median age at inclusion (in years), IQR	35 (25–40)
Active smoking at inclusion, *n*(%)	4 (16)
Median duration of Crohn’s disease, IQR	11 (3–19)
Median duration of perianal fistulizing Crohn’s disease, IQR	4 (2–8)
Mean follow-up time (in years)	3.7 (0.6)
Luminal disease at inclusion, *n*(%)	
None	21 (84)
Ileum	1 (4)
Proximal colon	1 (4)
Sigmoid	2 (8)
History of fistula surgery, *n*(%)	24 (96)
Number of previous fistula surgical intervention, IQR	3 (2–5)
Type of previous fistula surgery, *n*(%)	
Fistulotomy	2 (8)
Drainage of abscess with seton or drain placement	9 (38)
Drainage of abscess without drain placement	16 (64)
Seton placement	17 (68)
Laser treatment	1 (4)
Diverting colostomy at time of surgery, *n*(%)	1 (8)
Prior exposure to an anti-TNF agents, *n*(%)	23 (92)
CD medication at surgery, *n*(%)	22 (88)
Thiopurines	1 (4)
Anti-TNF agent	12 (48)
Combination therapy^a^	6 (24)
Ustekinumab	2 (8)
Vedolizumab	1 (4)
None	3 (12)
Number of external opening at time of surgery, *n*(%)	
1	10 (40)
2	10 (40)
3	5 (20)
TOpClass classification system at the time of surgery, *n*(%)	
Class 2a	16 (64)
Class 2b	7 (28)
Class 2c-i	1 (4)
Class 2c-ii	1 (4)
Complete clinical closure < 12 months, *n*(%)	13 (52)
Partial closure < 12 months, *n*(%)	20 (80)
Postoperative MRI (3 months postoperatively), *n*(%)	24 (96)
Radiologic healing at 3 months	9 (38)

Abbreviations: IQR, interquartile range; SD, standard deviation; TNF, tumor necrosis factor.

^a^Combination therapy is defined as thiopurine combined with an anti-TNF agent.

### Medical Treatment During Study Period

At the time of surgery, 18 patients were treated with an anti-TNF agent (72%) (12 patients on anti-TNF monotherapy, 6 patients received an anti-TNF agent and an immunomodulator). During the study period, no medical treatment change was observed in ten patients (56%), three patients switched to another anti-TNF agent (17%), one patient switched to another anti-TNF agent but stopped the medication on own initiative (5%), three patients switched to other biological(s) (17%) and one patient switched to another biological but was re-introduced to an anti-TNF agent (5%). Of those who were not treated with an anti-TNF agent perioperatively (*n* = 7) (28%), three patients (43%) were re-introduced on an anti-TNF agent during follow-up. During the study period, a medication switch was observed in 12/25 patients (48%).

### Long-Term Clinical Outcomes After Autologous PRS Injection

**Figure 2.**
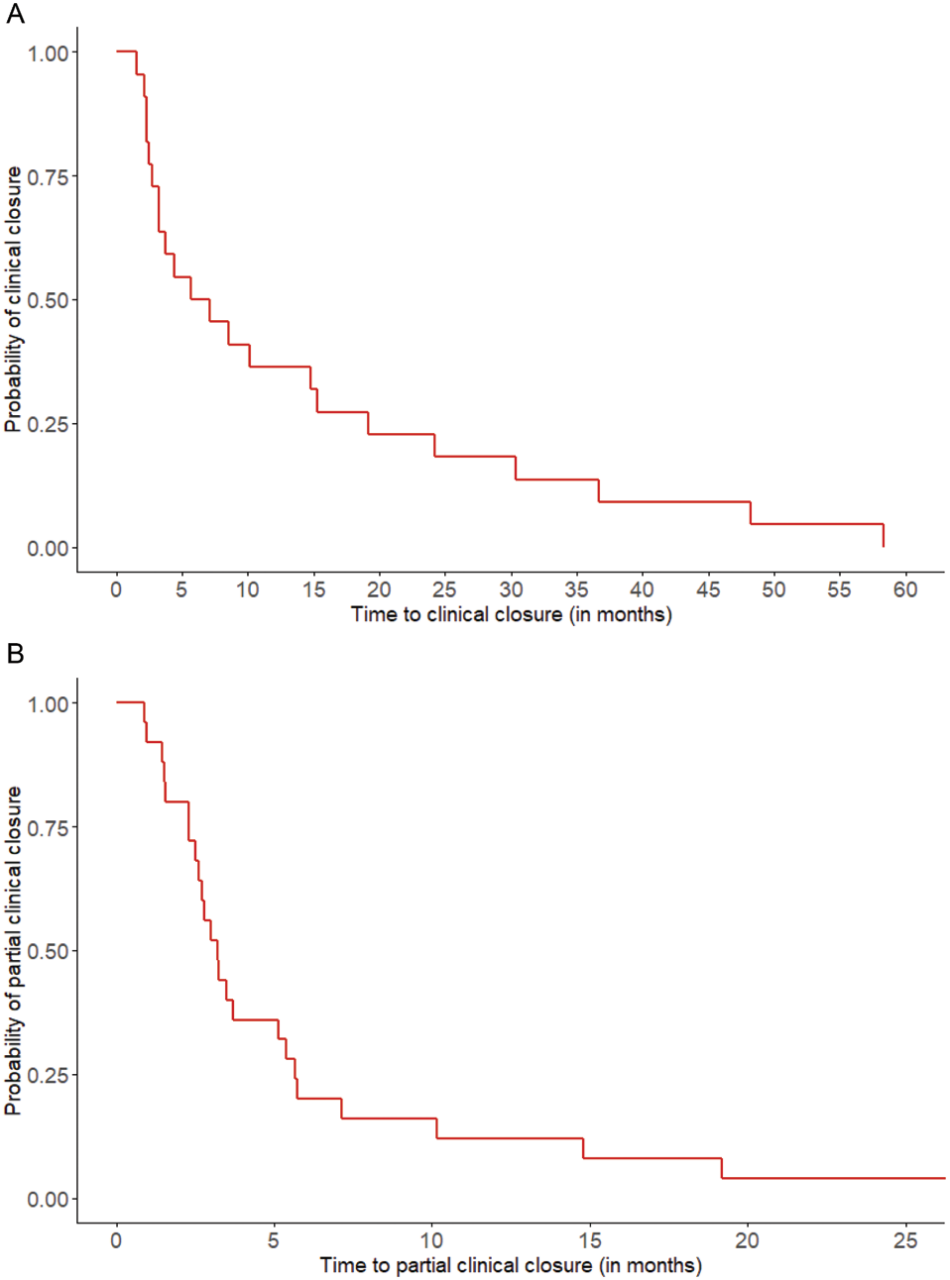
Survival probabilities for complete clinical closure (A) and partial clinical closure (B).

Within 1 year postoperatively, 13 patients (52%) reached complete clinical closure. Subsequently, complete clinical closure was achieved in another nine patients (36%) after this time period. Thus, complete clinical closure was achieved in 88% (*n* = 22) of the patients after a median time of 6.4 months (IQR 2.7–20.4) (Figure 2A) after the first treatment with additional autologous PRS injection, fistula curettage, and closure of the internal orifice. The three patients without complete clinical closure (12%) refrained from further treatment due to various reasons.

Within 1 year postoperatively, partial clinical closure was reached in 20 patients (80%). Partial clinical closure was observed in another five patients (20%) after 1 year postoperatively. Thus, all patients showed beneficial signs of clinical healing from fistula curettage and closure of the internal orifice in combination with PRS injection as partial clinical closure was achieved in all patients. The median interval between partial clinical closure and the first treatment with additional autologous PRS injection, fistula curettage, and closure of the internal orifice was 3.2 months (IQR 2.3–5.7) (Figure 2B). During the entire study period, recurrence was observed in two patients (8%) after reaching prior complete or partial clinical closure.

### Long-Term Radiologic Closure After PRS Injection

After the standardized first operative MRI at 3 months postoperatively, one (or more) MRIs were performed additionally in 19/24 (79%) patients with a median number of postoperative MRI scans of 4 (IQR 1–5) during the study period. Of the five remaining patients who did not undergo additional MRI at long-term follow-up, three patients had achieved radiologic healing at first postoperative MRI, and two patients wished no additional treatment. The modified van Assche Index decreased significantly from a median of 11 (IQR 9–16) points at baseline to 1 (IQR 1–3) points on the last available MRI (*P* < .001). At the last available MRI, radiologic healing was observed in 18 patients (75%). All patients with radiologic healing achieved complete clinical closure. Vice versa, patients with complete clinical closure achieved radiologic healing in 82% of the cases. After reaching radiologic healing, none of the patients experienced recurrence.

### Additional PRS Injections & Unplanned Re-Interventions During Follow-up

**Figure 3.**
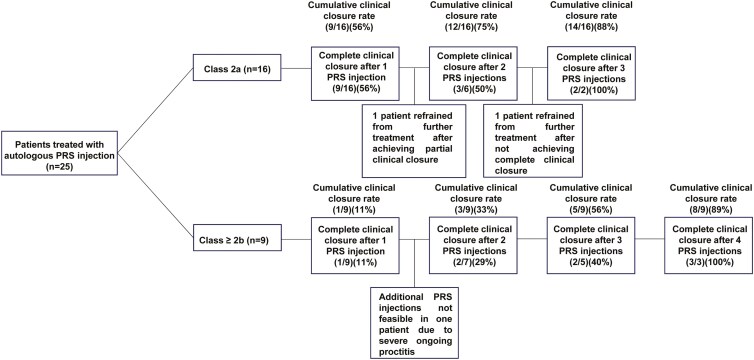
Clinical outcomes in subgroups according to the TOpClass classification system.^23^

During the study period, 13 patients (52%) were offered additional PRS injection(s) as an add-on to surgery (ie, fistula curettage and closure of the internal orifice) for either clinical and/or radiologic residual fistulizing disease (*n* = 12) or recurrent disease (*n* = 1). Of these 13 patients, six patients (46%) received one additional PRS injection, four patients (31%) received two additional PRS injections and three patients (23%) received three additional PRS injections. The rate of additional PRS injection combined with fistula curettage and surgical closure of the internal orifice was substantially lower in patients with a TOpClass classification system class 2a as compared to patients with a TOpClass classification system ≥ class 2b (38% vs. 78%, *P* = .05). The clinical outcomes and the need for additional PRS injections are displayed in Figure 3 for patients of both groups (TOpClass classification system class 2a and TOpClass classification system ≥ class 2b).

In addition, a total of 17 unplanned re-interventions were reported in 9/25 patients (36%) (median number of re-interventions 1; IQR 1–2) (3 patients in class 2a [33%], 6 patients in class 2b or higher [67%]). A detailed description of the unplanned re-interventions is displayed in Table 2. The indication for the unplanned re-interventions was for residual fistulizing disease (16/17) (94%) or recurrence (1/17) (6%). The rate of patients who underwent unplanned re-interventions was significantly lower in patients with a TOpClass classification system class 2a as compared to patients with a TOpClass classification system ≥ class 2b (19% vs. 67% *P* = .02).

**Table 2.** Overview of re-interventions during long-term follow-up.

Variables	Values
Number of patients with ≥1 re-intervention, *n*(%)	9 (36)
Median number of re-interventions, IQR	1 (1–2)
Type of re-interventions	
Incision and drainage alone	5
Incision and drainage with seton placement	4
Seton placement alone	4
Fistulotomy	2
Examination under anesthesia	1
Stoma formation with fistula curettage	1

## Discussion

The long-term outcomes of this uncontrolled pilot study showed that additional autologous PRS injection, fistula curettage, and closure of the internal orifice in patients with treatment-refractory pCD, treated in a tertiary referral center, is a promising treatment. All patients showed beneficial signs of clinical healing from fistula curettage and closure of the internal orifice in combination with PRS injection as partial clinical closure was achieved in all patients. Furthermore, complete clinical and radiologic healing was achieved in the majority of the patients. In addition, recurrence rates were low.

The use of additional PRS injection in combination with fistula curettage and closure of the internal orifice was introduced at first, in our tertiary referral center, as last-resort treatment for patients with refractory severe fistulizing disease in order to achieve symptom control or avoid ostomy and/or proctectomy surgery (class 2b or higher according to the TOpClass classification system).^21^ Although these patients are not considered to undergo surgical interventions aimed at fistula closure due to their disease severity, we observed that combined healing (ie, clinical closure and radiologic healing) rate was achieved in these patients (89%) at the end of follow-up in our study despite the high rates of additional PRS injections (78%) combined with fistula curettage and closure of the internal orifice.

As recommended by the current international guidelines, only surgical amendable patients (class 2a) should be offered combined medical and surgical treatment (anti-TNF agents with reconstructive fistula surgery) to achieve fistula closure.^5,6^ The outcomes of the PISA-II trial confirmed the superiority of combined medical and surgical treatment as compared to medical treatment alone on both short-term (<18 months) and long-term (median follow-up 5.7 years).^7,8^ The recommendation to only perform surgical interventions aimed at fistula closure in surgical amendable patients is supported by the findings of our study showing higher clinical closure rates, after one stage PRS injection with fistula curettage and closure of the internal orifice, in surgical amendable patients (class 2a) (56%) versus non-surgical amendable patients (≥ class 2b) (11%). We are aware that comparing our treatment results of this pilot study with the outcomes of other trials is difficult due to differences in study design, study population, interventions, and outcomes. However, when comparing the outcomes with the PISA-II trial, the outcomes of additional PRS injection as an add-on treatment are promising as higher closure and radiologic healing rates (88% vs. 72%; 75% vs. 42%), lower recurrence rates (13% vs. 22%) and comparable re-interventions rates (19% vs. 15%) were reported at long-term follow-up in patients classified as class 2a treated in our tertial referral center.^8^ Despite the promising results in this pilot study, randomized controlled trials are warranted to study the effects and the positioning of the additional PRS injection in the treatment for pCD. Futhermore, it is important to mention that the strict monitoring of these patients and the close collaboration between the treating gastroenterologist and surgeon may have contributed to these high success rates.

Radiologic evaluation with MRI is recommended as first-line diagnostic tool for the assessment of pCD and is considered to be superior compared to clinical evaluation.^24^ Furthermore, the MRI proved to be responsive to clinical improvement.^23,25^ However, it is still questioned when the first postoperative MRI should be performed as early clinical response may not necessarily correspond to obvious changes in MRI features.^22,26^ This issue is reflected by the moderate radiologic healing (38%) observed after a median interval of 3 months following PRS injection.^16^ Radiologic healing seems to be a valuable prognostic parameter for long-term fistula closure as no recurrences were observed in patients who achieved radiologic healing. This observation is in accordance with the outcomes of the PISA-II trial.^7,8^

The use of mesenchymal cells, derived from either bone marrow or adipose tissue (ASCs), to improve fistula healing has been assessed in several studies in recent years.^12–14,27–32^ To date, only three studies have reported long-term outcomes.^27,28,32^ Guillo et al. treated ten patients with a local injection of autologous SVF and microfat. At 3 years follow-up, remission defined as the absence of discharge of all external openings without any collections >2 cm on MRI, was achieved in seven patients.^27^ Barnhoorn et al. reported sustained clinical closure in 43%-100% in different groups treated with different concentrations of allogenic bone-marrow-derived mesenchymal stromal cells.^32^ Although comparing our results with these other long-term follow-up results is difficult due to differences in study design, study population, and outcomes, higher clinical closure rates were reported in our study. Long-term outcomes from the ADMIRE-CD study reported a clinical remission (clinical closure of all treated external openings) of 53% at three years follow-up in patients with darvadstrocel. No long-term outcomes on radiologic healing rates were reported.^28^ Despite the favorable outcome in the first ADMIRE-CD trial, the recent outcomes of ADMIRE-CD II phase III study, conducted in Europe, Israel, and North America, showed no significant differences at week 24 between patients treated with darvadostrocel versus placebo with regards to combined remission. Based on these outcomes, darvadstrocel has been recently withdrawn as a treatment option in Europe.^11,15^

A recently published review observed that SVF, isolated by mechanical fractionation similar to our procedure, seems to have a positive clinical effect in wound healing disorders (ie, perianal fistulas).^33^ The preservation of the extracellular matrix in mechanically isolated adipose-derived SVF is important due to its vital role in the regenerative properties of SVF. Extracellular matrix dictates cell proliferation, migration, differentiation, apoptosis or immunity, and angiogenesis regulation by binding and releasing paracrine factors in a controlled fashion.^18^ All these cellular processes are important in the regeneration of damaged tissue. These paracrine factors are mostly secreted by ASCs that act pro-angiogenic and anti-inflammatory. Moreover, extracellular matrix in SVF provides structural support to cells (eg, ASCs). Adipose-derived stromal cells reside as pericytes and supra-adventitial cells (precursor cell types) around vessels present in extracellular matrix.^34,35^ In this way, extracellular matrix most likely prevents ASCs from diffusion from the site of injection within the first hours. With regards to the use of autologous SVF in pCD, the pilot study of Dige et al. is the only study which assessed the effects of autologous adipose tissue for the treatment of pCD reporting a complete fistula healing rate of 57% (defined as no symptoms of discharge, no visible external fistula opening in the perineum, and no internal orifice detected by rectal digital examination). Complete resolution of the fistula tract, on pelvic MRI, was reported in 90% of the patients who achieved fistula healing.^12^ In addition, the same study research group reported an 86% healing rate in seven women treated with autologous adipose tissue for an anovaginal fistula (CD-related or radiation-induced). No data on radiologic healing were reported in this study.^36^ Further research in a large setting is warranted to assess the effects of SVF for the treatment of CD-related perianal, ano-, or rectovaginal fistulas.

Platelet-rich plasma contains a high concentration of growth factors that promote wound healing and neovascularization.^37^ Additional, certain chemokines in PRP are chemoattractant to ASCs.^38–41^ It is thought that PRP enhances production of paracrine factors by ASCs.^41^ However, whether the addition of PRP to SVF really leads to the potential increase in wound healing properties is still unknown. A recent study, performed in our center, did not report higher closure rates—on both short- and long-term—in patients with a transsphincteric cryptoglandular fistula who underwent transanal mucosal advancement flap (TAFR) with additional PRP injection as compared to patients treated with TAFR alone.^42^ In another study, we were able to demonstrate that injection of PRP combined with SVF clearly improved the outcome after flap repair in patients with a cryptoglandular fistula. However, it is questionable, whether this finding was due to an enhanced effect by combining both agents or to the action of SVF alone.^17^ Future studies are needed to assess the potential value of PRP to SVF.

This present study has several limitations. Firstly, this study is an explorative pilot study with a limited sample size and the absence of a control group. Secondly, no patient-reported outcomes were used as endpoint. Thirdly, the TOpClass classification system was retrospectively assessed in order to classify pCD severity at the time of first PRS injection by approximation.^21^ As this classification is subjective, an experienced colorectal surgeon, blinded for disease outcomes and the need for (unplanned) re-interventions, assessed the classification based on the individual charts and preoperative MRI perioperatively. At last, the composition of the PRS differs between individual patients, which may affect healing rates.^^43^^ Ongoing research is needed to study autologous cell therapy as an add-on to surgical treatments, including best-standardized composition and dose-response relation.

In conclusion, autologous PRS injection with fistula curettage and closure of the internal orifice is a promising therapy for patients with (treatment-refractory) pCD and could improve clinical and radiologic healing rates. In addition, low recurrence rates were observed. Future randomized research is warranted in order to assess the effectiveness and positioning of PRS in the field of pCD.
